# Effect of Laser Energy Density During LPBF on the Structure and Mechanical Properties of Al–15Sn–5Pb Alloy

**DOI:** 10.3390/ma18235268

**Published:** 2025-11-21

**Authors:** Nikolay M. Rusin, Alexander L. Skorentsev, Kirill O. Akimov, Vadim E. Likharev, Dmitry P. Il’yashchenko, Andrey I. Dmitriev

**Affiliations:** 1 Institute of Strength Physics and Materials Science SB RAS, 2/4 Akademicheskii Av., Tomsk 634055, Russia; rusinnm@mail.ru (N.M.R.); skoralexan@mail.ru (A.L.S.); akimov_ko@ispms.ru (K.O.A.); vel6@tpu.ru (V.E.L.); 2 School of Nuclear Science and Engineering, National Research Tomsk Polytechnic University, 30 Lenin Av., Tomsk 634050, Russia; 3 Department of Metal Physics, National Research Tomsk State University, 36 Lenin Av., Tomsk 634050, Russia; 4 School of Advanced Manufacturing Technologies, National Research Tomsk Polytechnic University, 30 Lenin Av., Tomsk 634050, Russia; 5 Yurga Technological Institute, National Research Tomsk Polytechnic University, 30 Lenin Av., Tomsk 634050, Russia; mita8@tpu.ru

**Keywords:** laser powder bed fusion (LPBF), volumetric laser energy density, critical energy density, aluminum matrix alloy, microstructure, ductility, compressive strength

## Abstract

Al–15Sn–5Pb (vol.%) alloy was fabricated by the Laser Powder Bed Fusion (LPBF) method at laser scanning speeds of 0.8, 1.0, and 1.2 m/s and laser powers ranging from 70 to 130 W. The samples were synthesized from a mixture of elemental powders using an ONSINT AM150 3D printer under a flowing argon atmosphere. The structure and mechanical properties under compression tests of the produced material were investigated as a function of the volumetric laser energy density (*E*) during LPBF. It has been established that low laser energy density during LPBF results in incomplete melting of aluminum particles and a non-uniform distribution of soft inclusions within the material. Increasing the energy density ensures a significantly more uniform distribution of the phases, resulting in the formation of a fine-grained three-phase alloy. It was established that both the ductility and strength of the alloy improve with the increase in *E* until a critical value is reached. As a result, at *E* ≥ 48 J·mm^−3^, the ultimate strength of the alloy reaches 100 ± 5 MPa, and its deformation before fracture is 15 ± 1%. Substituting one quarter of the tin volume with lead results in a significant increase in the ductility of the LPBF-fabricated aluminum alloy.

## 1. Introduction

Alloying aluminum with tin imparts antifriction properties due to the increased seizure pressure under dry and boundary friction. Such alloys find applications as antifriction materials in plain bearings and as phase change materials in thermal energy applications. This effect is caused by the ability of tin to be extruded onto the friction surface and to form a protective tribofilm [1,2,3,4,5,6,7,8,9,10]. According to the Russian standard GOST 14113-78 [11], the tin concentration in aluminum should be limited to 20 wt.% (~10 vol.%), since higher contents lead to the formation of continuous tin interlayers along the aluminum grain boundaries, which prevents their bonding into a strong load-bearing framework [12,13,14]. During plastic deformation, tin does not undergo strain hardening, and its interlayers in the loaded material act as areas of localized plastic flow; once incorporated into a shear band, these interlayers rapidly thin and fracture [14,15].

Therefore, the stronger the aluminum framework in such an alloy—that is, the lower the tin content—the higher the stress required to initiate plastic flow localization. For example, samples of the Al-20Sn-1Cu wt.% alloy (containing ~10 vol.% Sn) can withstand up to 30% tensile strain at an ultimate strength of 100–110 MPa. However, as shown in Ref. [16], an alloy with twice the tin content exhibiting comparable strength and high ductility can also be produced by sintering elemental Al and Sn powders. This alloy has a high seizure pressure under dry sliding against steel [14].

Since tin is insoluble in aluminum and is located between its grains, it could be expected that an increase in the specific surface area due to grain refinement would promote the formation of a stronger aluminum framework. However, experimental observations have shown that even at very high solidification rates of the Al–Sn melt, the precipitated aluminum solid-phase particles become surrounded by thin shells of liquid tin [17,18]. These interlayers, owing to their small thickness, have a limited reserve of ductility and rapidly fail once the flow stress reaches a certain level, which causes the fine-grained Al–20Sn (vol.%) alloy to exhibit high strength but low ductility.

It has been shown [3,19] that continuous tin interlayers can be fragmented into isolated inclusions if Al–Sn castings are subjected to severe plastic deformation followed by annealing. However, the shape and dimensions of the castings change considerably during such treatment, while their structure becomes coarser, making these methods economically inefficient for producing fine-grained materials. To prepare materials with a finely dispersed microstructure, the Laser Powder Bed Fusion (LPBF) process can be employed, which is characterized by extremely high heating and cooling rates of the deposited powder layer [20,21,22]. Nevertheless, as reported in [22,23], in Al–20Sn (vol.%) alloys produced by LPBF, a developed tin net still persists despite the reduced thickness of individual tin inclusions.

It was established in [24,25] that intergranular tin layers can also be disrupted by metallurgical means, namely by alloying Al–Sn systems with elements forming a eutectic with tin. For instance, lead—similar to tin—is insoluble in solid aluminum and, during cooling of the Al–Sn–Pb alloy, segregates to the periphery of aluminum grains as part of a liquid Sn–Pb film. This intergranular liquid layer decomposes at 183 °C into distinct tin and lead phases. The morphology of these intergranular regions depends on the volumetric Pb/Sn ratio. In Ref. [26], this ratio was 1:3, i.e., the volume fraction of tin in the melt was three times higher than that of lead. This Al–15Sn–5Pb alloy produced by LPBF, in which the total volume fraction of the soft phase (Sn + Pb) was equivalent to that in the Al–20Sn (vol.%) alloy, exhibited a lower strength but a threefold increase in ductility, which largely determines the wear resistance of antifriction materials used in sliding bearings [23]. Thus, the approach of improving ductility through the fragmentation of continuous intergranular tin layers proved to be effective. It should be noted that lead is less scarce than tin, and its addition significantly reduces the cost of the material. The mechanisms for improving the ductility of ternary LPBF alloys are not sufficiently studied, and their investigation is of great scientific interest.

Unfortunately, in the fabrication of this material, a narrow range of LPBF parameters was employed, differing only in the laser scanning speed (*v*) at a constant laser power of *P* = 70 W. Consequently, the volumetric energy density (*E*) delivered to a unit volume of the powder bed was limited [26]. This parameter can be increased, while keeping other LPBF parameters constant, only by increasing the laser power, since it is defined by the following relationship:

$$E\;=\;\frac{P}{\mathrm{hsv}},$$

where *h* is the powder layer thickness and *s* is the hatch spacing [27].

According to Ref. [23], at a laser power of *P* = 130 W and a scanning speed of *v* = 1.2 m/s, a volumetric energy density of approximately *E* ≈ 40 J·mm^−3^ was sufficient to produce Al–20Sn samples with maximum strength and low porosity. Further increases in *P* resulted only in a decrease in ultimate strength (σ_U_), while the material ductility remained low (ε ≤ 3.5%). In the Al–15Sn–5Pb alloy, in addition to tin, the presence of the much heavier lead requires additional time to achieve uniform concentration in the molten bath. During cooling of a homogeneous melt, it can be expected that solidification will yield a material with a more uniform phase distribution and improved mechanical properties. The time required for concentration equalization at a fixed *P* corresponds to LPBF regimes with lower laser scanning speeds. Therefore, the aim of this study is to investigate the structure and properties of the Al–15Sn–5Pb (vol.%) alloy produced over a wide range of laser scanning speeds at laser powers between 70 and 130 W.

## 2. Materials and Experimental Procedure

**Figure 1:**
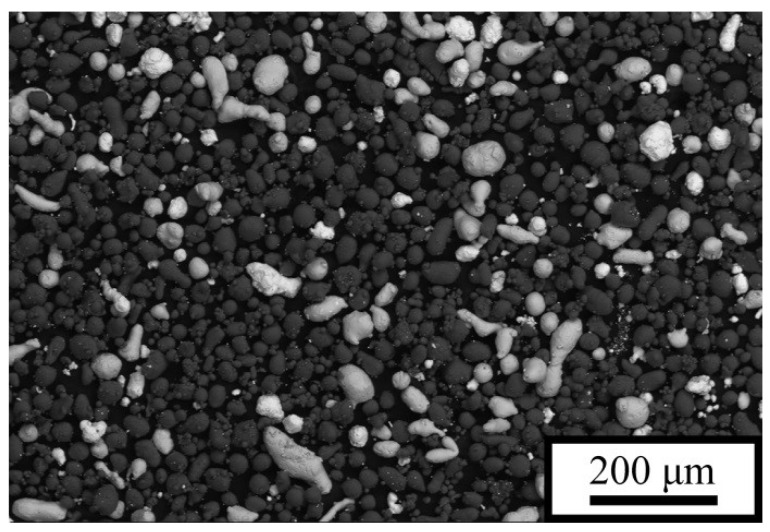
SEM image of the Al–15Sn–5Pb powder mixture. Bright particles correspond to Pb, gray particles to Sn, and dark particles to Al [26].

For the synthesis of the material by the LPBF method, pre-sieved elemental powders of aluminum ASD-1 grade [TU 48-8-226-87], tin PO-1 grade [28], and lead PS-1 grade [29] with particle sizes ranging from 25 to 50 μm were used. The powders were mixed in a Turbula-type mixer for 3 h at a container rotation speed of 30 rpm. A powder blend of the following composition (vol.%) was prepared: Al–15Sn–5Pb, hereafter referred to as P5 (Figure 1). The base composite of Al–20Sn (vol.%) composition is designated as P0. After mixing, the prepared powder mixtures were dried in a vacuum furnace at 120 °C for 3 h.

The samples were produced using an AM150 3D printer (ONSINT, Zelenograd, Russia). The LPBF process was carried out in a chamber filled with high-purity flowing argon, and the oxygen concentration in the chamber did not exceed 300 ppm. The thickness of the laser-irradiated powder layer was 30 μm, and this layer was applied to the surface of the synthesized sample with a squeegee. Each layer was deposited on top of the previous one onto a AMg6 alloy substrate preheated to 110 ± 5 °C using a rubber recoater blade. The laser scan strategy was linear with a 67° rotation between successive layers. The scanning speeds were 1.2, 1.0, and 0.8 m/s, the laser spot diameter was 75 ± 5 μm, and the hatch spacing was 90 μm. The laser power varied from 70 W to 130 W in 20 W increments.

The dimensions of the samples were 10 mm × 10 mm × 10 mm. The material porosity was determined by the hydrostatic weighing method in accordance with GOST 20018-74 [30]. Compression tests were performed using an Instron-1185 universal testing machine (Instron, Norwood, MA, USA) at a crosshead displacement rate of 0.5 mm/min. The specimens for compression were cut from the central region of the fabricated samples, with the loading axis oriented perpendicular to the LPBF build direction. Ends of the compressive specimens were lubricated with graphite prior to testing. For each LPBF processing condition, at least three specimens were tested.

Metallographic specimens were prepared using a standard procedure: grinding with SiC abrasive papers of progressively decreasing grit size, followed by polishing on a cloth pad using a diamond suspension containing particles smaller than 1 μm. The polished samples were then cleaned in an ultrasonic bath with alcohol.

The microstructure of the polished specimens was examined using a LEO EVO 50 scanning electron microscope (Carl Zeiss, Oberkochen, Germany) equipped with an energy-dispersive X-ray spectroscopy (EDS) attachment. X-ray diffraction (XRD) data were obtained using a DRON-8H diffractometer (Innovation Center Burevestnik, Saint Petersburg, Russia) with Cu Kα radiation (λ = 1.5406 Å) over a 2θ range of 10–140°. The diffraction patterns were processed using the PDWin V3.0 software package (Innovation Center Burevestnik, Saint Petersburg, Russia).

## 3. Results and Discussion

**Figure 2:**
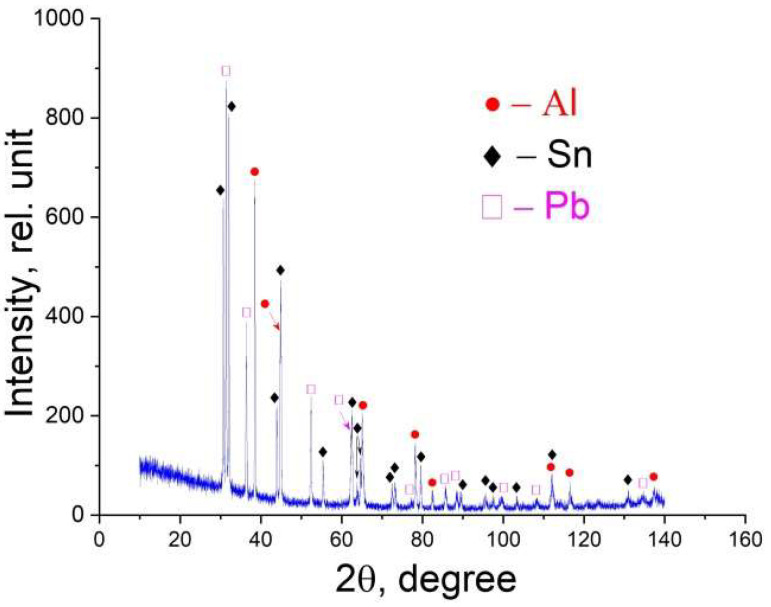
X-ray diffraction pattern of the P5 alloy produced at *v* = 0.8 m/s and *P* = 70 W.

From the given image of the initial powder mixture, it is evident that its components are distributed relatively evenly, which indicates that the mixing conditions were chosen correctly (Figure 1). The samples synthesized by the LPBF method consisted of three phases—Al, α-Sn, and β-Pb (Figure 2)—in full agreement with the equilibrium phase diagram. The amount of energy transferred to a unit volume of the powder bed is presented in Table 1. As follows from the data, despite the different combinations of *P* and *v*, some LPBF regimes produced samples with similar values of *E*. This indicates that if *E* is the dominant LPBF parameter governing the structure and, consequently, the mechanical properties of the fabricated composites, then the deformation behavior of samples produced at comparable *E* values can be expected to be similar. A total of twelve types of samples were fabricated: two at *E* ≈ 26–28 J·mm^−3^, three at *E* ≈ 32–34 J·mm^−3^, three at *E* ≈ 40–42 J·mm^−3^, and two at *E* ≈ 48–51 J·mm^−3^. Additionally, single samples were produced at the minimum (*E* = 21.6 J·mm^−3^) and maximum (*E* = 60 J·mm^−3^) energy densities.

**Figure 3:**
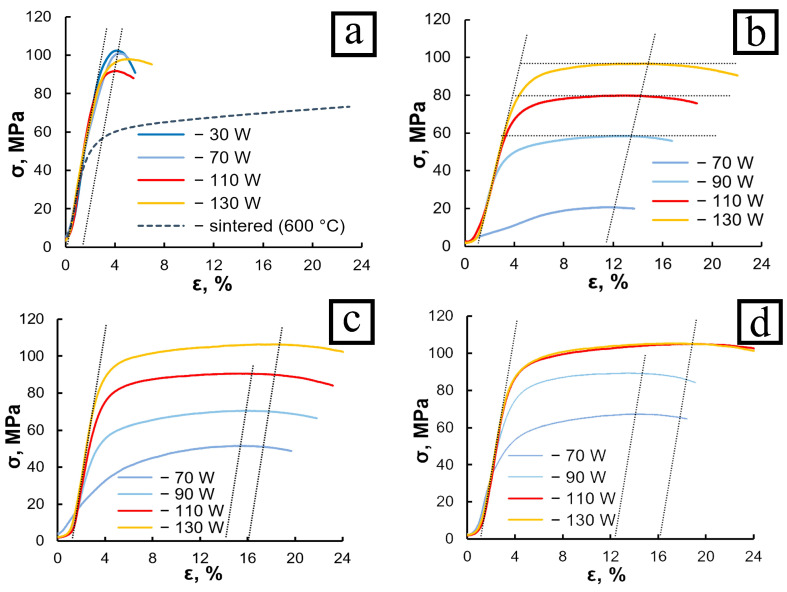
Compressive stress–strain curves of the P5 alloy fabricated by the LPBF method at laser scanning speeds (m/s): 1.2 (**a**,**b**), 1.0 (**c**), and 0.8 (**d**). Alloy compositions: P0 (**a**) and P5 (**b**–**d**).

Figure 3 shows the corresponding compressive stress–strain curves for the fabricated samples. For comparison, the compression curves of LPBF-fabricated samples with composition P0 produced under the same processing conditions are also presented. As seen from the plots, the value σ_U_ of P0 alloy is in the range of 100–105 MPa and decreases slightly with increasing laser power. The ductility of the samples also improves marginally under these conditions (Figure 3a).

A comparison of the plots in Figure 3a,b shows that under identical LPBF processing conditions and with the same volume fraction of the aluminum phase, the σ(ε) curves of the P0 and P5 alloys differ significantly. For the P0 alloy, the segment of the curve following the elastic region is very short: the flow stress σ rapidly reaches its maximum value σ_U_ at a strain of approximately ε ≈ 1.5% and then sharply decreases due to material fracture. Increasing the laser power results in a slight reduction in the strength of the P0 alloy and a moderate increase in ductility up to ε ≈ 3%. Thus, grain refinement in the P0 alloy leads to an increase in flow stress accompanied by a pronounced loss of ductility. The decrease in ductility of LPBF-fabricated alloys [23,26] is attributed to a sharp decrease in the ductility reserve of the intergranular tin layers as they become thinner. To support this conclusion, Figure 3a also shows the compression curve (dashed line) of a sample of the same composition produced by vacuum sintering. It can be seen that plastic flow of the sintered sample begins at relatively low stresses and continues even after ε > 20%, while the material has not yet reached its maximum strength σ_U_.

The shape of the compression curves for the P5 alloy differs from those of P0 by the greater length of the plastic flow region (Figure 3b). The maximum strength σ_U_ of the P5 samples is shifted toward higher strains, reaching ε = 11–12%. The strongest sample was prepared at *E* = 40 J·mm^−3^, with its σ_U_ nearly matching that of the P0 alloy produced at the same energy density. The strength of P5 samples fabricated at lower *E* values was lower, although their maximum ductility remained high (ε = 11–12%).

A decrease in the laser scanning speed up to *v* = 1.0 m/s led to an increase in the volumetric energy density *E*. The samples produced at this scanning speed exhibited slightly higher compressive strength, with the maximum value shifted along the strain axis to approximately ε ≈ 14% (Figure 3c). Thus, reducing *v* resulted in a slight improvement in ductility and an increase in σ_U_, primarily due to a higher yield stress σ_0.2_ and more pronounced strain hardening of the material. The strengthening effect was especially notable for samples fabricated at lower *E* values.

A further decrease in the scanning speed to v = 0.8 m/s led to an additional increase in the flow stress of the samples. In this case, the σ(ε) curves of the specimens produced at laser powers of *P* = 130 and 110 W nearly coincided (Figure 3d). According to Table 1, these samples were fabricated at energy densities exceeding *E* > 48 J·mm^−3^. Therefore, it can be assumed that this value of *E* represents a critical threshold for the P5 composition, and that its further increase—either by raising *P* or by reducing *v*—does not have a significant effect on the mechanical properties of the synthesized samples (Figure 3).

Thus, it can be concluded that substituting one quarter of the tin volume with lead in the LPBF-fabricated Al-20Sn alloy resulted in a significant improvement in its ductility. The alloy strength, however, decreased, especially at energy densities *E* < 40 J·mm^−3^. Increasing *E* to approximately 48 J·mm^−3^—either by raising *P* or by reducing *v*—provided samples with the highest combination of mechanical properties.

**Figure 4:**
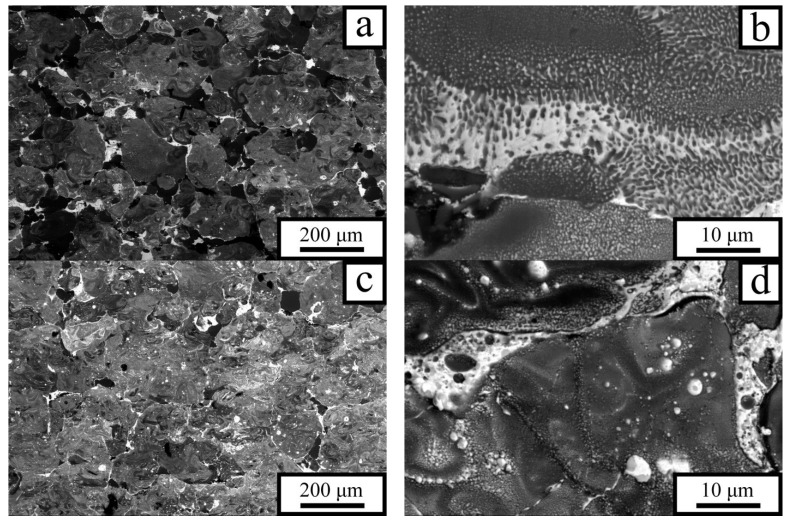
SEM images of the P5 alloy structure fabricated by LPBF at a laser power of *P* = 70 W and laser scanning speeds of *v* = 1.2 m/s (**a**,**b**) and *v* = 0.8 m/s (**c**,**d**).

Since the influence of the energy density *E* on the mechanical properties of the P5 alloy is manifested through the characteristics of its resulting microstructure, the corresponding structural features were investigated. Figure 4 presents microstructural images of the P5 alloy produced at a laser power of 70 W and *v* = 1.2 m/s (*E* = 21.6 J·mm^−3^) and *v* = 0.8 m/s (*E* = 32.4 J·mm^−3^). That is, in this case, a decrease in *v* led to an increase in *E* by ≈50%.

The alloy produced at the lowest energy density *E* exhibits a heterogeneous phase distribution throughout the material volume. Moreover, in addition to the phases precipitated from the solidifying melt, regions of unmolten primary aluminum are present (dark particles in the micrographs, Figure 4a). These are presumably aluminum powder particles covered with a thick oxide film. Adjacent to them are gray regions with a eutectic structure, representing domains of Al–Sn alloy (Figure 4b). The samples also contained a small number of pores, the proportion of which was 1–2% and was practically independent of the laser energy at *E* > 25 J·mm^−3^. The features of tin distribution indicate that the alloy formation during laser heating of the powder layer proceeded as follows: neighboring aluminum powder particles melted and coalesced into larger regions, while liquid tin and lead were located along their peripheries. Tin began to diffuse into the aluminum regions, enriching the upper layers of the molten aluminum with Sn, whereas the concentration decreased with depth. Thus, the upper areas of the molten aluminum regions consisted of a tin-enriched liquid, the intermediate layers contained less Sn, and the central regions were occupied by molten aluminum that had not yet mixed with tin. The undissolved tin was distributed between aluminum particles and mixed with lead, which precipitated as discrete particles upon cooling of the Sn–Pb melt. Hence, the relatively low temperature of molten aluminum and the presence of solid particles hindered the development of convective flow, so mixing with alloying elements occurred mainly through mutual diffusion.

The incident energy density *E* increases with decreasing laser scanning speed, which strongly affects the microstructure of the P5 alloy. The number of unmolten aluminum particles decreases markedly, while the fraction of gray regions—representing Al–Sn mixture areas—increases (Figure 4c). The size and number of Sn–Pb inclusions diminish as tin dissolves into aluminum, leading to a more uniform distribution of tin throughout the material. As seen in Figure 4d, convective flows begin to play an increasingly important role in the mixing of liquid tin with aluminum, alongside diffusion. These flows transport tin into the larger aluminum regions more efficiently than diffusion alone. The tin-enriched regions formed in this way are clearly visible in Figure 4d as light-contrast boundaries. A portion of the tin remains trapped inside the rapidly solidifying aluminum regions as isolated inclusions. Lead does not mix appreciably with aluminum and is located within the tin inclusions situated along the outer boundaries of the large aluminum regions.

**Figure 5:**
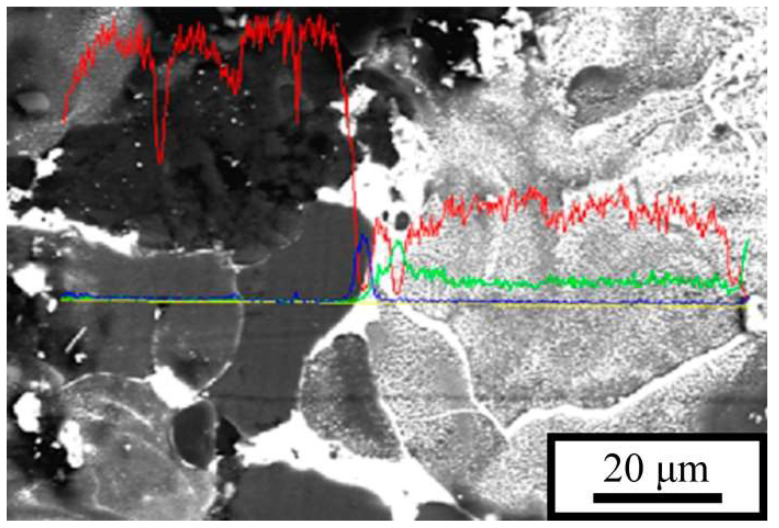
Microstructure of the P5 alloy and the corresponding elemental distribution. LPBF parameters: *P* = 70 W, *v* = 1 m/s. Elemental intensity profiles: aluminum (red), tin (green), and lead (blue). Electron beam scan line is marked by yellow.

This elemental distribution is confirmed by the image shown in Figure 5, where the intensity profiles of the elemental reflections are superimposed on the microstructural section along the scan line. The image reveals that there are regions of pure aluminum as well as eutectic regions with a constant Al/Sn ratio in the P5 alloy fabricated at low *E*. During solidification, all regions of the melt are divided into large fragments separated by thin tin interlayers. Lead, in contrast to tin, is practically insoluble in aluminum and is located in the intergranular regions together with tin; during solidification of the melt, it precipitates as discrete particles.

**Figure 6:**
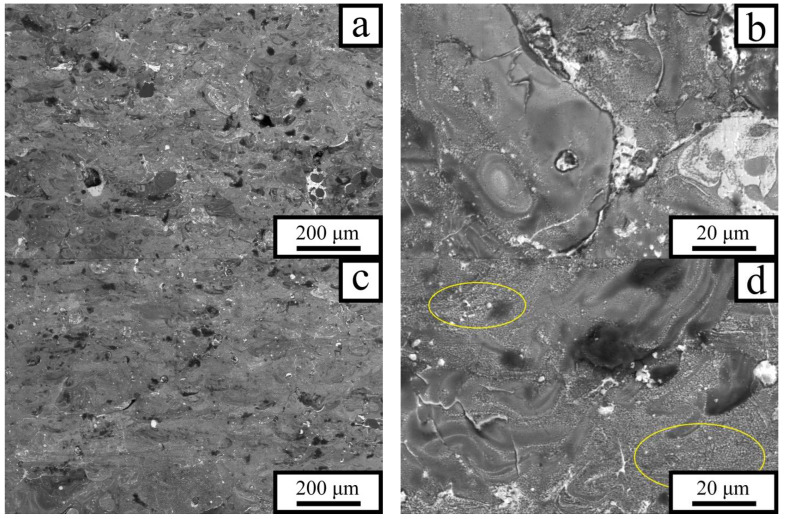
Microstructure of the P5 alloy. Laser power *P* = 130 W; laser scanning speeds *v* = 1.2 m/s (**a**,**b**) and 0.8 m/s (**c**,**d**).

The microstructure of the samples produced at the same laser scanning speeds but at a higher laser power of *P* = 130 W is shown in Figure 6. A comparison with the previous micrographs reveals that the structure of the alloy fabricated at higher laser power is more homogeneous than that of the alloy produced at *P* = 70 W. Even in the sample prepared at *v* = 1.2 m/s (*E* ≈ 40 J·mm^−3^), almost no unmolten or poorly mixed regions remain (Figure 6a), and the tin inclusions—both large and small—observed in Figure 4 and Figure 5 as thin surface layers have nearly disappeared. Relatively fine intergranular inclusions corresponding to the Sn–Pb eutectic can be seen only at higher magnifications (Figure 6b).

Figure 6c,d show the microstructure of the samples exhibiting the best mechanical performance—high ductility combined with high strength. This is a consequence of the fact that the inclusions of soft phases are isolated and do not form a continuous net. Compared with the previous samples, this structure is even more homogeneous, with almost no tin segregations in the form of intergranular layers. No areas of unmelted aluminum were detected. The main part of the material volume is occupied by regions (outlined in yellow in Figure 6d) where aluminum is uniformly mixed with tin. However, some elongated regions with a compositional gradient of tin are still present; during solidification, tin was expelled toward their peripheries. Such regions are much more numerous and larger in Figure 6b than in Figure 6d, which explains the lower mechanical properties of the samples (Figure 3d).

**Figure 7:**
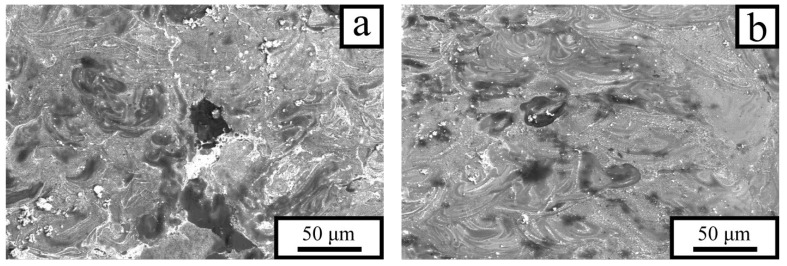
Microstructure of the P5 alloy. Laser power *P* = 110 W; laser scanning speeds *v* = 1.2 m/s (**a**) and 0.8 m/s (**b**).

A structure similar to the relatively homogeneous one observed in Figure 6d is also formed at *P* = 110 W, provided that the incident energy density on the sample surface exceeds 48 J·mm^−3^. In this case, due to the high melt temperature, mixing of the components occurs predominantly through multiple convective flows. During solidification, a fine-grained structure is formed in which tin does not have sufficient time to produce flat inclusions by diffusion (Figure 7b) that would otherwise promote localized plastic flow. As a result, the strain distribution within the loaded sample remains relatively uniform, contributing to the simultaneous increase in both ductility and strength of the material (Figure 3d).

Thus, the energy density *E* has a strong influence on the microstructure of the P5 alloy and, consequently, on its mechanical properties. When *E* exceeds 48 J·mm^−3^, numerous regions with a homogeneous distribution of tin in aluminum, which prevents deformation localization in the loaded material, are formed during the solidification. In this case, both the strength and ductility of the alloy are governed primarily by the mechanical behavior of the aluminum matrix. However, when *E* is insufficient to ensure intensive and uniform mixing of tin and aluminum, solidification results in the formation of regions containing planar tin inclusions (Figure 7a), which reduce the flow stress of the samples (Figure 3). In this case, lead acts as a beneficial additive, as its particles within the tin interlayers hinder the formation of extended bands of localized shear.

## 4. Conclusions

Based on the obtained results from the study of the structure and mechanical properties of the Al–15Sn–5Pb alloy fabricated by the LPBF method under various laser irradiation regimes, the following conclusions can be drawn:

- Substituting one quarter of the tin volume with lead results in a significant increase in the ductility of the LPBF-fabricated Al–15Sn–5Pb (vol.%) alloy. Therefore, a promising direction for further research is the study of Al-20(Sn + Pb) (vol.%) alloys with higher lead content.
- The strength of the Al–15Sn–5Pb (vol.%) alloy depends on the degree of compositional homogeneity, which is determined by the intensity of melt mixing. The higher the volumetric laser energy density *E* delivered to the powder bed, the more intensive the mixing of components and the higher the strength of the LPBF-fabricated ternary alloy.
- Increasing *E* beyond 48 J·mm^−3^ has practically no significant effect on the mechanical properties of the Al–15Sn–5Pb (vol.%) alloy.

## Figures and Tables

**Table 1:** Dependence of the volumetric energy density *E* (J·mm^−3^) on the LPBF processing parameters.

Scanning Speed *v*, m/s	Laser Power *P*, W			
	70	90	110	130
1.2	21.6	27.8	34.0	40.1
1.0	25.9	33.3	40.7	48.1
0.8	32.4	41.6	50.9	60.1

## Data Availability

The original contributions presented in this study are included in the article. Further inquiries can be directed to the corresponding author.
